# Targeting IGF-1R: throwing out the baby with the bathwater?

**DOI:** 10.1038/sj.bjc.6606023

**Published:** 2011-01-04

**Authors:** B Basu, D Olmos, J S de Bono

**Affiliations:** 1 Drug Development Unit, The Royal Marsden NHS Foundation Trust and The Institute of Cancer Research, Downs Road, Sutton, Surrey SM2 5PT, UK

There are intense commercial pressures in industry to develop drugs for large unselected populations, although this remains a risky and expensive strategy. Several examples now exist where targeted treatments are utilised in molecularly defined cancer patient populations. The EGFR tyrosine kinase inhibitor (TKI) gefitinib is a case in point, failing to show a clear benefit in non-small-cell lung cancer (NSCLC) patients when given with first-line chemotherapy. Gefitinib has nevertheless re-emerged as an important therapeutic following the confirmation that mutations in the TK domain of EGFR confer sensitivity to it (Mok *et al*, 2009), with evidence that this population is enriched within Asian, female and never-smoker patients with adenocarcinoma (Lynch *et al*, 2004; Paez *et al*, 2004). More recently, two large-phase III trials, investigating the addition of the fully human monoclonal antibody (mAb) to the insulin-like growth factor 1 receptor (IGF-1R) figitumumab (CP-751,871, Pfizer) to carboplatin/paclitaxel (ADVIGO 1016) and to the EGFR TKI erlotinib (ADVIGO 1018), in advanced NSCLC patients have been suspended after planned interim analyses indicated futility (Jassem *et al*, 2010). These data raise several important questions: Was there sufficient evidence to support these phase III trials? Could we have learnt more from early-phase data to identify the patients who are most likely to benefit? Is IGF-1R a key target in NSCLC? How should we design our trials to identify predictive biomarkers that decrease the risk of such late and costly failures?

Gualberto and colleagues now publish valuable data in this edition of *The British Journal of Cancer* (Gualberto et al, 2010b) evaluating putative predictive circulating biomarkers of sensitivity to figitumumab. Their study highlights the complexities of predictive biomarker clinical qualification. They conclude that, independent of tumour characteristics, pre-treatment free IGF-1 (fIGF-1) concentration is a predictive biomarker of clinical benefit from figitumumab at 20 mg kg^−1^ but not at 10 mg kg^−1^ in NSCLC. Their results are consistent with observations that low IGF-1 levels are associated with prolonged survival in NSCLC (Han *et al*, 2006). Nevertheless, their finding that higher baseline fIGF-1 is present in females and patients with adenocarcinoma is at odds with reported data indicating that patients with squamous cell carcinoma derive more benefit from figitumumab and calls into question whether this is simply a prognostic biomarker (Karp *et al*, 2009a). Importantly, however, in this manuscript under discussion, pre-treatment fIGF-1 was not predictive of PFS in patients receiving chemotherapy alone, suggesting that this may not be simply a prognostic biomarker. Recent reports profiling molecular determinants of sensitivity to figitumumab also identified increased IGF-1R expression within squamous cell tumours, which were more likely to respond, but could not definitively establish whether this was a prognostic or predictive factor (Gualberto *et al*, 2010).

Overall, analysis of these data is complicated by the small sample size and the biological heterogeneity of patients on trial, which are common issues in such clinical research. Their use of one-sided tests limits the statistical power and calls into question whether this study is adequately powered. Moreover, the addition of chemotherapy renders the determination of biomarkers that are truly figitumumab-specific more complex. Other factors that cannot be underestimated are measures of the analytical validity of the assay. Overall, evaluation of the reproducibility and variability of the assay by using two baseline readings should be recommended for such studies. Indeed, concerns remain that current assay methodologies to measure IGF-1 bioactivity are controversial and imperfect (Frystyk, 2007).

Despite these criticisms, these attempts to detect circulating predictive biomarkers are to be commended. We are convinced that circulating predictive biomarkers are critically important in cancer research; these are repeatable, less invasive and more easily implemented in large randomized trials. Nonetheless, the relationship between circulating biomarkers and tumour characteristics must be analysed to evaluate whether these reflect tumour biology. Moreover, pre-treatment biomarkers provide a ‘snapshot’ suggesting which patients may benefit from treatment, but repeated analyses are required to establish a picture of adaptive changes through acquired resistance mechanisms. Indeed, earlier phase I trials evaluating figitumumab reported that treatment was associated with increased circulating IGF-1 levels and decreased soluble IGF-1R from baseline (Lacy *et al*, 2008; Molife *et al*, 2010). This supports repeated analyses of such biomarkers, which is best done through circulating biomarkers. Importantly, the feedback increase in IGF-1 post treatment with figitumumab may explain why the higher dose of 20 mg kg^−1^ is more active than the 10 mg kg^−1^ dose.

Figitumumab phase I trial data suggested that bioactive IGF-1 levels may influence treatment sensitivity following the observation of responses in patients treated with figitumumab at doses above 10 mg kg^−1^ who had a high baseline free fIGF-1 to IGF-binding protein-3 (IGF-BP3) ratio (Karp *et al*, 2009b). In a phase II, randomised NSCLC trial of first-line paclitaxel/carboplatin (PC) alone or in combination with figitumumab (PCF), the combination resulted in an impressive overall response rate (ORR) of 54% (Karp *et al*, 2009a). Intriguingly, there was an apparent dose response to figitumumab in both ORR and 12-week PFS in patients with adenocarcinoma and squamous cell carcinoma histology, with the greatest benefit seen with the higher antibody dose of 20 mg kg^−1^ (78% ORR and 89% 12-week PFS). Moreover, anti-tumour activity was observed in two patients with squamous histology receiving figitumumab monotherapy after PC discontinuation for progression. Despite this, no patient stratification or population enrichment based on histological subtype, IGF-1R tumour expression or circulating fIGF1 levels was pursued in Phase III trials (Jassem *et al*, 2010).

Several questions remain; deregulation of the IGF signalling axis in NSCLC is supported by findings that increased IGF-1 and low levels of its binding protein IGF-BP3 are associated with an increased risk of lung cancer (Yu *et al*, 1999; Han *et al*, 2006). Furthermore, IGF-1R is frequently over-expressed in NSCLC, mediating signalling that results in tumour growth and drug resistance (Morgillo *et al*, 2007). However, IGF-1-overexpressing transgenic mice with functionally upregulated IGF-1R are predisposed towards increased formation of adenomata but not malignant tumours, whereas preclinical work indicates that IGF-II may instead be the critically important autocrine/paracrine ligand in NSCLC by also signalling via the insulin receptor (IR) (Quinn *et al*, 1996; Ulanet *et al*, 2010). It remains to be seen whether treatments targeting both IGF-1R and IR, or both IGF-1 and IGF-II, in NSCLC will yield different results (Olmos *et al*, 2010a). Nonetheless, we have observed impressive anti-tumour activity of figitumumab in metastatic Ewing's sarcoma as a single agent, with some patients experiencing durable responses up to 3 years, suggesting that targeting IGF-1R alone deserves further evaluation (Olmos *et al*, 2010b).

Finally, several different strategies can be pursued to gain most information from early-phase studies. These include phase I trial expansions, phase II Bayesian adaptive designs where all-comers are initially treated, but patients are then enriched for ‘responding phenotypes’ as in the Biomarker-Integrated Approaches of Targeted Therapy for Lung Cancer Elimination (BATTLE) clinical trial programme, and randomised phase II trials with either *a priori* selection of patients with or without the presence of the biomarker or, as in this case, a retrospective analysis of putative biomarkers against outcome from treatment. Overall, however, we urgently need to develop smarter trial designs that can accelerate the clinical qualification of putative predictive biomarkers in concert with targeted drug trials to expedite the successful delivery of less costly drug approval and patient benefit. Although the initial and NSCLC trials of figitumumab have been negative, the evaluation of drugs targeting the IGF pathway should continue. We should not throw out the baby with the bathwater.

## References

[bib1] Frystyk J (2007) Utility of free IGF-I measurements. Pituitary 10(2): 181–187 17429595 10.1007/s11102-007-0025-y

[bib2] Gualberto A, Dolled-Filhart M, Gustavson M, Christiansen J, Wang YF, Hixon ML, Reynolds J, McDonald S, Ang A, Rimm DL, Langer CJ, Blakely J, Garland L, Paz-Ares LG, Karp DD, Lee AV (2010) Molecular analysis of non-small cell lung cancer identifies subsets with different sensitivity to insulin-like growth factor I receptor inhibition. Clin Cancer Res 16(18): 4654–4665 20670944 10.1158/1078-0432.CCR-10-0089 PMC2952544

[bib3] Gualberto A, Hixon ML, Karp DD, Li D, Green S, Dolled-Filhart M, Paz-Ares LG, Novello S, Blakely J, Langer CJ, Pollak MN (2010b) Pre-treatment levels of circulating free IGF-1 identity NSCLC patients who derive clinical benefit from figitumumab. Br J Cancer 104: 68–74 21102589 10.1038/sj.bjc.6605972 PMC3039819

[bib4] Han JY, Choi BG, Choi JY, Lee SY, Ju SY (2006) The prognostic significance of pretreatment plasma levels of insulin-like growth factor (IGF)-1, IGF-2, and IGF binding protein-3 in patients with advanced non-small cell lung cancer. Lung Cancer 54(2): 227–234 16935391 10.1016/j.lungcan.2006.07.014

[bib5] Jassem J, Langer CJ, Karp DD, Mok T, Benner RJ, Green SJ, Park K, Novello S, Strausz J, Gualberto A (2010) Randomized, open label, phase III trial of figitumumab in combination with paclitaxel and carboplatin versus paclitaxel and carboplatin in patients with non-small cell lung cancer (NSCLC). J Clin Oncol (Meet Abstr) 28(Suppl 15): 7500

[bib6] Karp DD, Paz-Ares LG, Novello S, Haluska P, Garland L, Cardenal F, Blakely LJ, Eisenberg PD, Langer CJ, Blumenschein Jr G, Johnson FM, Green S, Gualberto A (2009a) Phase II study of the anti-insulin-like growth factor type 1 receptor antibody CP-751,871 in combination with paclitaxel and carboplatin in previously untreated, locally advanced, or metastatic non-small-cell lung cancer. J Clin Oncol 27(15): 2516–2522 19380445 10.1200/JCO.2008.19.9331

[bib7] Karp DD, Pollak MN, Cohen RB, Eisenberg PD, Haluska P, Yin D, Lipton A, Demers L, Leitzel K, Hixon ML, Terstappen LW, Garland L, Paz-Ares LG, Cardenal F, Langer CJ, Gualberto A (2009b) Safety, pharmacokinetics, and pharmacodynamics of the insulin-like growth factor type 1 receptor inhibitor figitumumab (CP-751,871) in combination with paclitaxel and carboplatin. J Thorac Oncol 4(11): 1397–1403 19745765 10.1097/JTO.0b013e3181ba2f1d PMC2941876

[bib8] Lacy MQ, Alsina M, Fonseca R, Paccagnella ML, Melvin CL, Yin D, Sharma A, Enriquez Sarano M, Pollak M, Jagannath S, Richardson P, Gualberto A (2008) Phase I, pharmacokinetic and pharmacodynamic study of the anti-insulinlike growth factor type 1 receptor monoclonal antibody CP-751,871 in patients with multiple myeloma. J Clin Oncol 26(19): 3196–3203 18474873 10.1200/JCO.2007.15.9319

[bib9] Lynch TJ, Bell DW, Sordella R, Gurubhagavatula S, Okimoto RA, Brannigan BW, Harris PL, Haserlat SM, Supko JG, Haluska FG, Louis DN, Christiani DC, Settleman J, Haber DA (2004) Activating mutations in the epidermal growth factor receptor underlying responsiveness of non-small-cell lung cancer to gefitinib. N Engl J Med 350(21): 2129–2139 15118073 10.1056/NEJMoa040938

[bib10] Mok TS, Wu YL, Thongprasert S, Yang CH, Chu DT, Saijo N, Sunpaweravong P, Han B, Margono B, Ichinose Y, Nishiwaki Y, Ohe Y, Yang JJ, Chewaskulyong B, Jiang H, Duffield EL, Watkins CL, Armour AA, Fukuoka M (2009) Gefitinib or carboplatin-paclitaxel in pulmonary adenocarcinoma. N Engl J Med 361(10): 947–957 19692680 10.1056/NEJMoa0810699

[bib11] Molife LR, Fong PC, Paccagnella L, Reid AH, Shaw HM, Vidal L, Arkenau HT, Karavasilis V, Yap TA, Olmos D, Spicer J, Postel-Vinay S, Yin D, Lipton A, Demers L, Leitzel K, Gualberto A, de Bono JS (2010) The insulin-like growth factor-I receptor inhibitor figitumumab (CP-751,871) in combination with docetaxel in patients with advanced solid tumours: results of a phase Ib dose-escalation, open-label study. Br J Cancer 103(3): 332–339 20628389 10.1038/sj.bjc.6605767 PMC2920021

[bib12] Morgillo F, Kim WY, Kim ES, Ciardiello F, Hong WK, Lee HY (2007) Implication of the insulin-like growth factor-IR pathway in the resistance of non-small cell lung cancer cells to treatment with gefitinib. Clin Cancer Res 13(9): 2795–2803 17473213 10.1158/1078-0432.CCR-06-2077

[bib13] Olmos D, Basu B, de Bono JS (2010a) Targeting insulin-like growth factor signaling: rational combination strategies. Mol Cancer Ther 9(9): 2447–2449 20807783 10.1158/1535-7163.MCT-10-0719

[bib14] Olmos D, Postel-Vinay S, Molife LR, Okuno SH, Schuetze SM, Paccagnella ML, Batzel GN, Yin D, Pritchard-Jones K, Judson I, Worden FP, Gualberto A, Scurr M, de Bono JS, Haluska P (2010b) Safety, pharmacokinetics, and preliminary activity of the anti-IGF-1R antibody figitumumab (CP-751,871) in patients with sarcoma and Ewing's sarcoma: a phase 1 expansion cohort study. Lancet Oncol 11(2): 129–135 20036194 10.1016/S1470-2045(09)70354-7 PMC2941877

[bib15] Paez JG, Janne PA, Lee JC, Tracy S, Greulich H, Gabriel S, Herman P, Kaye FJ, Lindeman N, Boggon TJ, Naoki K, Sasaki H, Fujii Y, Eck MJ, Sellers WR, Johnson BE, Meyerson M (2004) EGFR mutations in lung cancer: correlation with clinical response to gefitinib therapy. Science 304(5676): 1497–1500 15118125 10.1126/science.1099314

[bib16] Quinn KA, Treston AM, Unsworth EJ, Miller MJ, Vos M, Grimley C, Battey J, Mulshine JL, Cuttitta F (1996) Insulin-like growth factor expression in human cancer cell lines. J Biol Chem 271(19): 11477–11483 8626706 10.1074/jbc.271.19.11477

[bib17] Ulanet DB, Ludwig DL, Kahn CR, Hanahan D (2010) Insulin receptor functionally enhances multistage tumor progression and conveys intrinsic resistance to IGF-1R targeted therapy. Proc Natl Acad Sci USA 107(24): 10791–10798 20457905 10.1073/pnas.0914076107 PMC2890766

[bib18] Yu H, Spitz MR, Mistry J, Gu J, Hong WK, Wu X (1999) Plasma levels of insulin-like growth factor-I and lung cancer risk: a case-control analysis. J Natl Cancer Inst 91(2): 151–156 9923856 10.1093/jnci/91.2.151

